# Spectroscopic and Theoretical Analysis of the Interaction between Plasma Proteins and Phthalimide Analogs with Potential Medical Application

**DOI:** 10.3390/life13030760

**Published:** 2023-03-10

**Authors:** Edward Krzyżak, Aleksandra Kotynia, Dominika Szkatuła, Aleksandra Marciniak

**Affiliations:** 1 Department of Basic Chemical Sciences, Faculty of Pharmacy, Wroclaw Medical University, Borowska 211a, 50-556 Wrocław, Poland; 2 Department of Medicinal Chemistry, Faculty of Pharmacy, Wroclaw Medical University, Borowska 211, 50-556 Wrocław, Poland

**Keywords:** fluorescence spectroscopy, circular dichroism spectroscopy, IR spectroscopy, plasma proteins, phthalimide derivatives

## Abstract

One of the groups of organic compounds with potential use in medicine and pharmacy is phthalimide derivatives. They are characterized by a wide range of properties such as antibacterial, antifungal, and anti-inflammatory. In this study, we focused on research on four phthalimide derivatives with proven non-toxicity, which are cyclooxygenase inhibitors. With the use of molecular docking study and spectroscopic methods, such as fluorescence, circular dichroism, and FT-IR spectroscopies, we analyzed the way the tested compounds interact with plasma proteins. Among the many proteins present in the plasma, we selected three: albumin, α1-acid glycoprotein, and gamma globulin, which play significant roles in the human body. The obtained results showed that all tested compounds bind to the analyzed proteins. They interact most strongly with albumin, which is a transport protein. However, interactions with serum albumin and orosomucoid do not cause significant changes in their structures. Only in the case of gamma globulins significant changes were observed in protein secondary structure.

## 1. Introduction

Phthalimide analogs are a very interesting group of compounds from a pharmaceutical point of view. These molecules have many biological activities, such as antibacterial, anticonvulsant, anti-inflammatory, or antifungal [1,2]. Their use and their importance in medicine were described in our previous work about N-substituted 1H-isoindole-1,3(2H)-dione derivatives [3]. We have shown there that newly tested imides are characterized by a good affinity for both isoforms of cyclooxygenase and are non-toxic. Therefore, we decided to continue our research.

In the study of pharmacodynamics and pharmacokinetics of new therapeutic agents, the analysis of the interaction between plasma proteins and drugs is very important [4]. The information about the binding of drugs with proteins allows for defining the concentrations of free and bound forms of the pharmaceuticals [5]. The liquid fraction of blood is composed, inter alia, of plasma proteins, such as albumin, α1-acid glycoprotein, antibodies, fibrinogen, or globulins [4,6]. Therefore, we decided to investigate the interaction of phthalimide derivatives with some of the proteins mentioned.

Serum albumin is the main protein responsible for transport in the living organism. The distribution and metabolism of pharmaceuticals are correlated with their affinities toward this protein [7]. Drugs can bind with albumins (with both BSA—bovine serum albumin, and HSA-human serum albumin) with the formation of stable complexes. In this study, we select the BSA as a model protein. Its stability, low cost, and structure similar to HSA make it can be successfully used in this type of research. The α1-acid glycoprotein (AAG) is the second protein we selected. It is the acute phase protein. The concentration of AAG changes during infections, in pregnancy, or with the use of drugs [4,8]. The molecule of AAG is negatively charged in neutral pH. It can bind and transport lots of basic and neutral pharmaceuticals in the human body [9]. Therefore, the study of the interaction of new potential drugs with this protein is crucial, as in the case of albumin. The last analyzed protein is gamma globulin (GG). It plays an important role in the immune system. The GG contains a few species of immunoglobulins which are responsible, inter alia, for the identification and neutralization of foreign objects, such as bacteria and viruses [8]. Therefore, the study of the interaction with the three proteins mentioned will be an excellent next step in the analysis of the biodistribution of the new phthalimide analogs.

**Figure 1:**
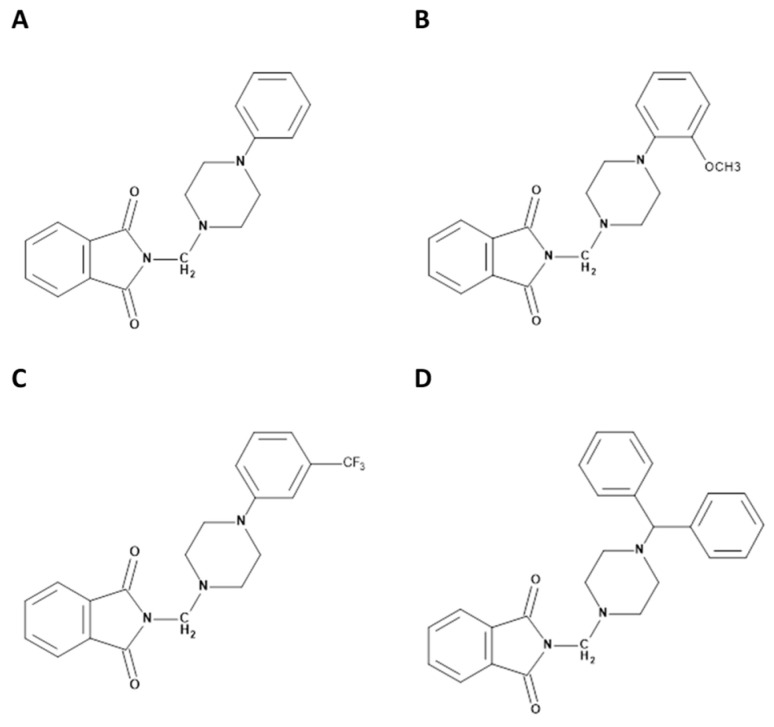
The structures of analyzed compounds A, B, C, and D.

In this study, we have chosen four phthalimide derivatives: A, B, C, and D (Figure 1) for analysis, previously described in work [3]. The final products A–D were obtained by aminoalkylation of the acidic proton on the imide nitrogen atom of the isoindoline-1,3-dione. The reaction was carried out for several hours using aqueous formaldehyde and the appropriate arylpiperazine under reflux tetrahydrofuran. Final products A–D were obtained in good (47.26%-C, 58.92%-D) or very good (76.87%-A; 92.91%-B) yields. Spectral studies of FT-IR, NMR (1H, C13) as well as elemental and mass analysis MS/MS and ESI-MS confirmed the structure and homogeneity of the obtained derivatives, which was supplemented by a fragmentation study identifying the presence of molecular quasi-ions. To investigate the interaction between analyzed N-Substituted 1H-isoindole-1,3(2H)-dione analogs and proteins, we have used several spectroscopic methods such as UV–Vis spectroscopy, circular dichroism (CD) spectroscopy, fluorescence spectroscopy, and FT-IR spectroscopy. Furthermore, all analyzed interactions will also be investigated using a molecular modeling study. All theoretical and analytical methods used will allow for determining the way the tested phthalimide analogs interact and whether they bind to plasma proteins.

## 2. Materials and Methods

### 2.1. Chemicals

The synthesis of compounds analyzed was performed in the Department of Medicinal Chemistry, Wroclaw Medical University, and described in work [3]. Studied proteins, BSA, AAG, GG, and 0.01 M phosphate buffer tablets were bought from Sigma-Aldrich Chemie GmbH, (St. Louis, MO, USA). 

### 2.2. Spectroscopic Studies

#### 2.2.1. Fluorescence Spectroscopy

Cary Eclipse 500 spectrophotometer (Agilent, Santa Clara, CA, USA) was used to measure fluorescence spectra. The 3D fluorescence experiments were performed at excitation and an emission wavelength of 200–350 nm in steps of 5 nm and 250–500 nm, respectively. The concentrations of BSA, AAG, and GG were 1.0 μM. We used 3 mL of a solution of each protein and we added a small portion of 1.0 mM phthalimide analogs. Experiments were performed at 3 values at temperatures of 297, 303, and 308 K in pH = 7.4, in phosphate buffer as a solvent. The parameters of measurements of the quenching spectra were as follows: 300 nm for excitation, 300–500 nm emission wavelength, and 10 mm path length. The molar ratio of compound to protein was 0.1–2.0 with 0.2 steps for BSA and GG, and 1–10 with 1.0 steps for AGG. Furthermore, for BSA binding studies, we used the two site markers, Phenylbutazone (PHB, site I marker) and Ibuprofen (IBP, site II marker). Concentrations of protein and markers in this experiment were equal to 1.0 μM and 3.0 μM, respectively.

#### 2.2.2. Circular Dichroism Spectroscopy

The Jasco J-1500 magnetic circular dichroism spectrometer (JASCO International CO., Tokyo, Japan) was used to measure CD spectra. Circular dichroism spectra were collected for protein solutions and after adding small portions of analyzed phthalimide analogs. Phosphate buffer was used as a solvent here (pH 7.5), and due to this, physiological parameters were simulated here. The parameters of measurements of CD measurements were as follows: the range was 205–250 nm for BSA and AAG, and 210–250 nm for GG, scan speed rate was equal to 50 nm/min, with a response time of 1 s, path length—10 mm. The concentrations of solution used were 1.0 μM, and 1.0 μM for the proteins and phthalimide analogs, respectively. The molar ratios of proteins and ligands were equal to 1:0, 1:0.5, 1:1, 1:5, and 1:10. The CD Multivariate Calibration Creation and CD Multivariate SSE programs (JASCO International CO., Tokyo, Japan) were used for the analysis of the secondary structure elements. Mean residue molar concentrations of proteins have been included in this analysis.

#### 2.2.3. FT-IR Measurement

The Nicolet iS50 FT-IR (Thermo Fisher Scientific, Waltham, MA, USA) was used to collect spectra. The spectrophotometer was equipped with a deuterated triglycine sulfate (DTGS) detector, and KBr beam splitter, and Attenuated Total Reflectance (ATR) accessory. All spectra were measured at room temperature of 297 K and data were recorded within 3000 to 600 cm^−1^ with a wavelength step of 4 cm^−1^, and 100 scans were averaged for each spectrum. Protein stock solution with a concentration equal to 1.0 mM for BSA, concentration equal to 0.1 μM for AAG, and GG were prepared in medium phosphate buffer. The concentration of the studied compounds was 10 mM for measuring BSA interaction and 0.1 mM for measuring AAG and GG interaction. The appropriate small value of the compound solution was added to 200 µL solution of protein to achieve 0.5, 1.0, 1.5, and 2.0 molar ratios. Each solution mixture was dropped on an ATR crystal, and the spectrum was recorded. The secondary structure analysis was kept out by Omnic 9.3.30 software (Thermo Fisher Scientific Inc.) which is dedicated to the spectrophotometer.

### 2.3. Molecular Docking

The mode of interactions between compound A–D and plasma proteins was calculated by the molecular docking method. First, the geometry of the tested compounds was optimized. Density Functional Theory was applied. B3LYP/6-311+G (d.p) was used as a basis set [10,11,12]. Computation was performed by Gaussian 2016 A.03 software package [13]. The experimentally determined 3D structure of proteins was downloaded from RCBS Protein Data Bank. The following structures were used for modeling: 3V03 (bovine serum albumin), 2BXC and 2BXG (human serum albumin), 3KQ0 (α1-acid glycoprotein), 1AJ7 (gamma globulin). To prepare the ligands and receptors, input file AutoDock Tools 1.5.6 [14] was used. From protein structures, co-crystallized molecules of ligands and water were eliminated. Kollman partial charges and non-polar hydrogens have also been added. The compounds A-D were prepared by the standard procedure: rotatable bonds were ascribed, nonpolar hydrogens were merged, and partial charges were added. The main docking parameters were defined as Lamarckian Genetic Algorithm with 100 running times and 2.5 million evaluation times. The grid box was determined based on the active site with size 60 × 60 × 60 and 0.375 Å spacing. The study of interaction was discovered by AutoDock v.4.2.6. After docking, the results were analyzed and visualized using Discovery Studio Visualizer v.20.

## 3. Results and Discussion

### 3.1. Molecular Docking Studies

Firstly, to determine whether analyze phthalimide analogs can bind to BSA, AAG, and GG, a theoretical simulation was constructed. The calculated results of Binding Free Energy (Δ*G*°) are listed in Table 1. As is well known, the more negative the value, the stronger the interactions. The energy was in the range of −27.42–−37.91 kJ·mol^−1^. This is sufficient to form a stable complex. 

In the BSA molecule, there are two binding sites with a high affinity for drug binding. Site I is situated in subdomain IIA and site II is situated in subdomain IIIA [15]. The molecular docking simulation showed that both active sites are accessible for compounds A–D. However, it is preferable for them to be located in a hydrophobic pocket in the subdomain IIIA. The difference is rather small. The strongest interaction was detected for the BSA-D system (−37.91 kJ·mol^−1^), i.e., compounds with two benzene rings. The results also indicate that the substituent -OCH_3_ in the ortho position (B) and -CF_3_ in the meta position of the aromatic ring (C) slightly decrease the stability of the complex.

**Figure 2:**
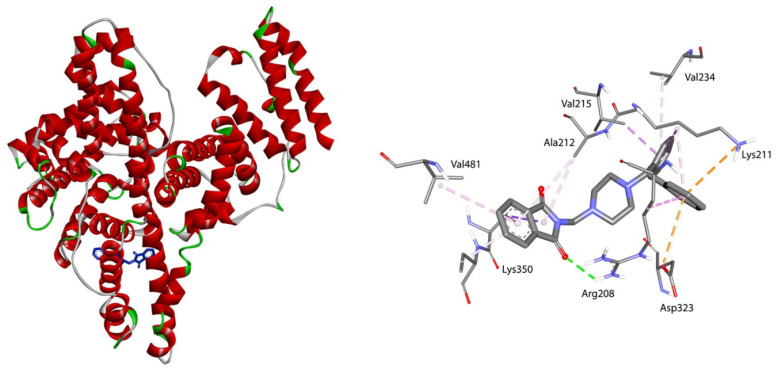
On the left—the location of phthalimide derivative D (blue) in subdomain IIIA; on the right—the 3D view of the binding mode.

**Figure 3:**
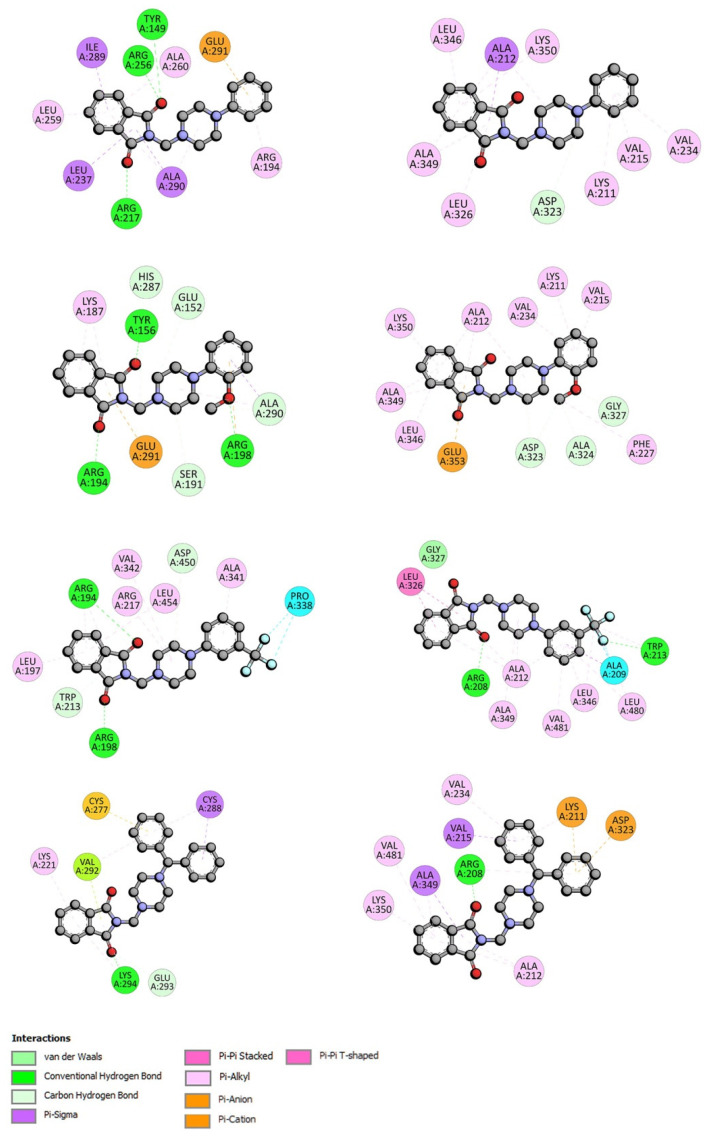
The plane diagram of a type of interaction between phthalimide derivatives and Bovine Serum Albumin. On the left in site I, and on the right in site II.

The location of phthalimide derivative D in the bonding cavity of drug site II is shown in Figure 2. Compound D interacts with BSA by hydrogen bonds between Arg208 and the oxygen atom from the carbonyl group. The isoindoline-1,3-dione moiety is surrounded by Ala349, Ala212, Lys350, and Val481. π-σ and π-alkyl contacts are detected. A set of two benzene rings forms hydrophobic interactions with Val215 by π-σ contact, with Val234, Lys211, and Arg208 by π-alkyl, and with Lys211 (π-cation) and Asp232 (π-anion). For compounds A-C, all parts of the molecule, isoindoline-1,3-dione part, piperazine ring, and phenyl group interact with BSA in site II. An illustration of a type of interaction is presented in Figure 3 (right). In site I, hydrogen bonds play an important role with oxygen atom from the carbonyl group. Compound A binds to BSA via Tyr144, Arg217, Arg256; compound B binds by Tyr156, Arg194, Arg198; compound C binds by Arg148, Arg194; and compound D binds by Lys294. All complexes are additionally stabilized by hydrophobic and Van der Waals contacts (Figure 3, left).

**Figure 4:**
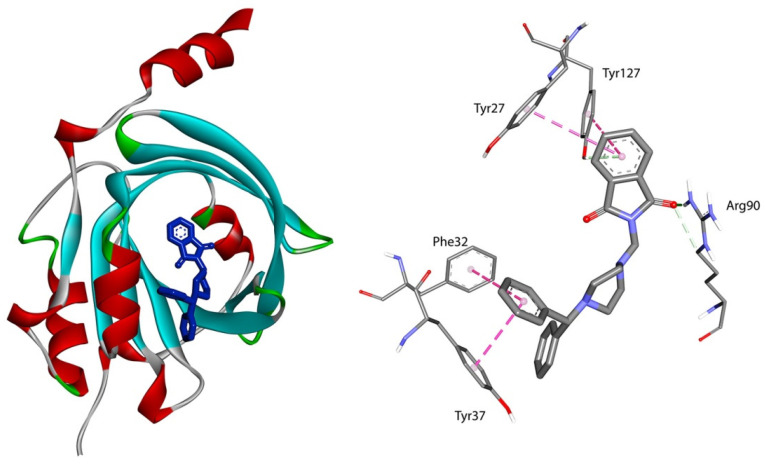
On the left—the pose of phthalimide derivative D (blue) in the AAG active pocket; on the right—the 3D view of binding mode.

**Figure 5:**
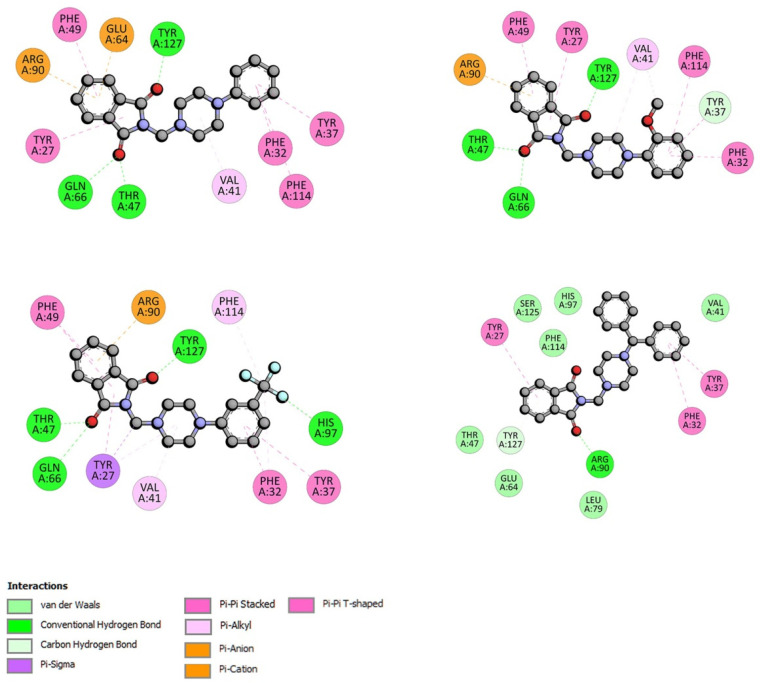
The plane diagram of a type of the interaction between phthalimide derivatives and α1-acid glycoprotein.

For interactions with AAG, structural modifications of compound A result in stronger interactions. Adding a substituent in the phenyl (compounds B, C) slightly lowers the Δ*G*°. Replacing one benzene ring with two (compound D) quite significantly lowers the Δ*G*° (Table 2). The orientation of compound D in the active pocket of AAG is given in Figure 4. The -C=O group is entangled in a hydrogen bond with Arg90 residue. Hydrophobic interaction, π-π stacked or π-π T-shaped, between benzene rings and Tyr27, Tyr37, and Phe32 is observed. For the complexes with phthalimide derivatives A–C, three hydrogen bonds are observed, between Thr47, Gln66, Tyr127, and two carbonyl groups from isoindoline-1,3-dione moieties. Hydrophobic and Van der Waals contacts increase the stability of the complex (Figure 5). 

**Figure 6:**
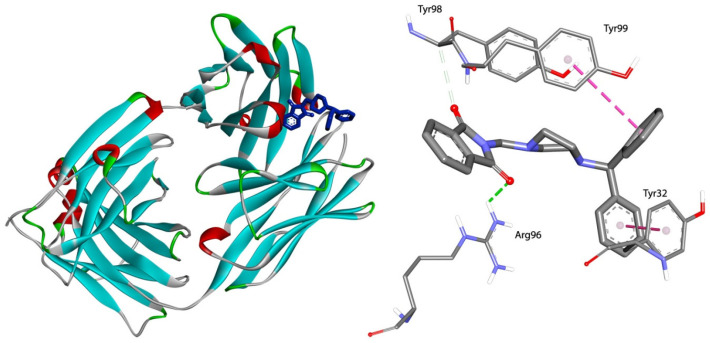
On the left—the position of phthalimide derivative D (blue) in GG active pocket; on the right—the 3D view of binding mode.

**Figure 7:**
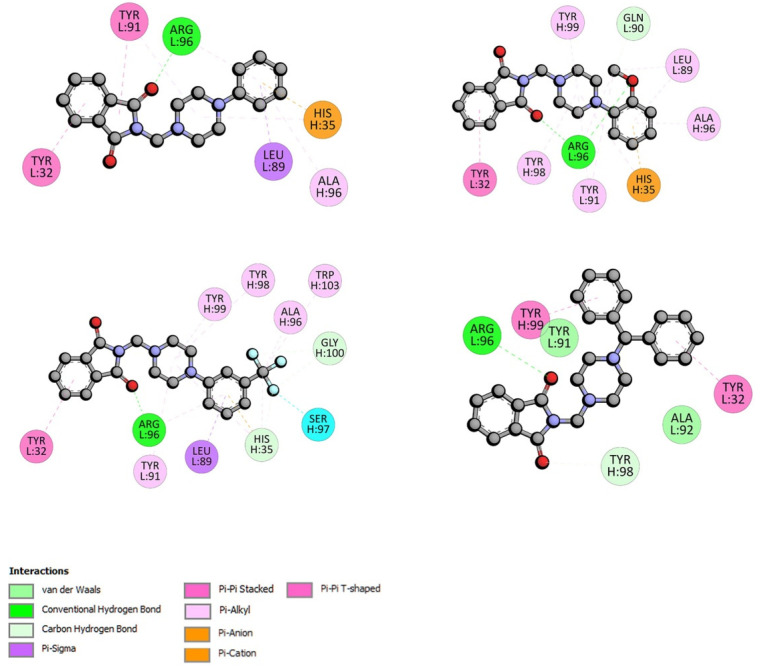
The plane diagram of a type of interaction between phthalimide derivatives and gamma globulin.

The docking analysis indicated that the strongest interaction with gamma globulin is observed for phthalimide derivatives C and D, −35.09 and −35.12 kJ·mol^−1^, respectively. The location of compound analog D in the active site of GG is presented in Figure 6. Only one hydrogen bond between Arg96 and the oxygen atom from the carbonyl group is observed. The complex is further stabilized by hydrophobic interactions π-π stacked and π-π T-shaped between Tyr32, Tyr99, and two benzene rings. The Arg98 residue forms hydrogen bonds with the carbonyl group also in complexes with A, B, and C. The π contacts are observed. The details are presented in Figure 7.

Due to its high structural similarity with human serum albumin, BSA has widely been used as a model protein for studying the binding interaction between drugs and serum albumin. To show that the studied compounds interact in a similar way with HSA, additionally, molecular docking was also performed on human serum albumin. The molecular docking results are presented in Table 1. For both drug sites, the Binding Free Energy is negative. Site II is slightly preferred, similar to interaction with BSA. In addition, the most stable is the complex with phthalimide D. In site I, hydrogen bonds play an important role with oxygen atom from the carbonyl group with Arg, Lys, Gln residues. In site II, hydrogen bonds are formed by Arg, Leu, Val. Several hydrophobic interactions stabilize the complexes. The details of the interactions are shown in Figure S2 in Supplementary Files.

Because from theoretical results it can be concluded that all analyzed compounds formed complexes with proteins studied, and observed differences are not significant, all four phthalimide derivatives were subjected to experimental studies.

### 3.2. Fluorescence Spectroscopy

#### 3.2.1. Fluorescence of Compounds A–D

**Figure 8:**
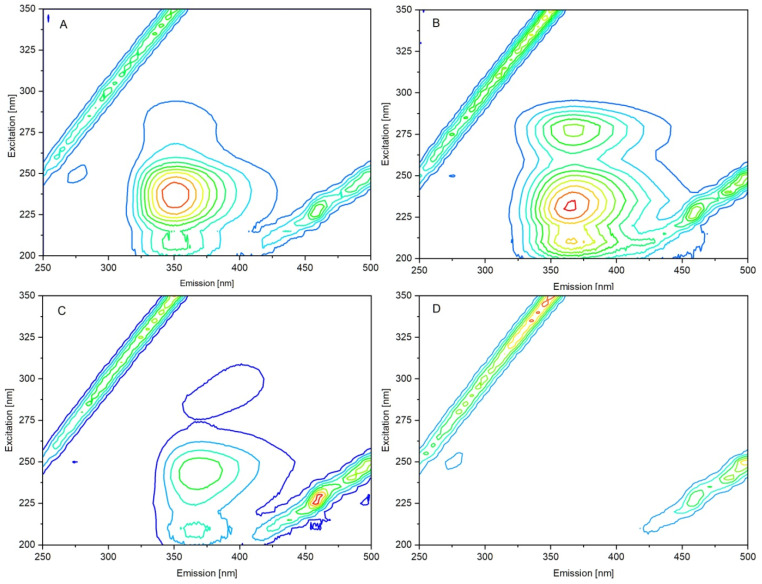
3D fluorescence spectra (as contour plots) of compounds A–D. Fluorescence intensity increases from blue to red.

To determine the fluorescence behavior of studied compounds in phosphate buffer solution, a three-dimensional fluorescence spectroscopy measurement was performed. The spectra were recorded with excitation wavelengths set to 200–350 nm, and emission wavelengths set to 250–500 nm. Obtained contour plots are presented in Figure 8. As it has been shown, compounds A, B, and C have fluorescence properties in the studied excitation range. The λ_ex_/λ_em_ were determined as 237/351, 232/367, and 243/370 for A, B, and C, respectively. Compound D showed no fluorescence properties.

In this work, we investigated the interactions between phthalimide derivatives, promising COX inhibitors, with plasma proteins serum albumin, α-1 acid glycoprotein, and gamma globulin, to confirm the pharmaceutical potential of studied compounds. The main technique was a fluorescence quenching study after adding the tested compound to the protein probe. 

As it is commonly known, the fluorescence of proteins is related to the presence of tryptophan (Trp) and tyrosine (Tyr) residues (and slightly phenylalanine). The Trp has a maximum of excitation at 280 nm and emission from approximately 305 to 350 nm, depending on solvent polarity [16]. The absorption spectrum of Tyr largely overlaps with Trp, and emission is less sensitive to solvent polarity with a maximum of approximately 300–305 nm. As is shown in Figure 8, at an excitation of 280 nm, the emission spectrum of A–C is significant. To avoid overlapping peaks, we searched for the excitation wavelength at which the fluorescence from Trp or Tyr was still significant and the intrinsic fluorescence of A–C was not observed (or was negligible). We determined that such conditions are met by excitation at a wavelength of 300 nm (Figure 8). If the emission spectrum is measured at 300 nm, the studied compounds are not exited under these conditions. Tyrosine residues are also not exited, but the fluorescence of tryptophans can still provide information on protein conformational transitions [17]. 

#### 3.2.2. Fluorescence Quenching of BSA, AAG, and GG by Compounds A, B, C, D

**Figure 9:**
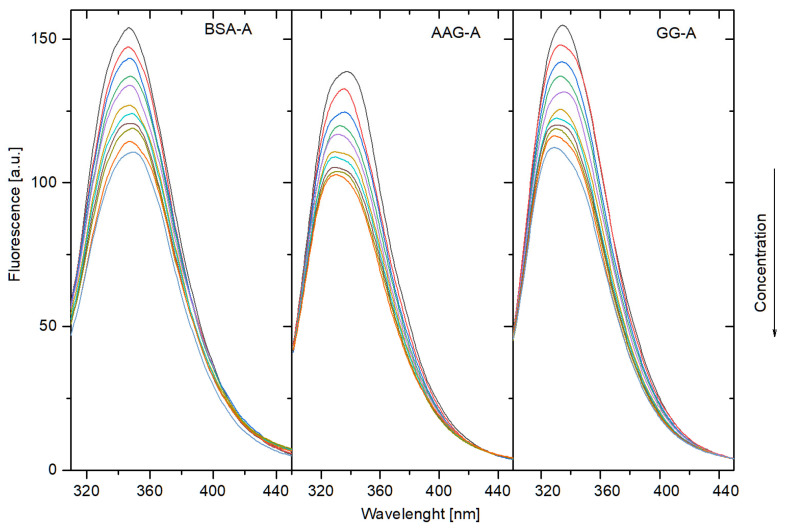
Fluorescence spectra BSA, AAG, and GG in the presence of different concentrations of compound A.

To determine the nature of the interaction of the tested compounds with plasma proteins, fluorescence quenching was used. It is a simple, widely used method leading to good and reliable results. Due to the intrinsic fluorescence of compounds A–C and overlapping emission spectra with Trp and Tyr residues, as mentioned above, the excitation wavelength was selected as 300 nm. Bovine serum albumin has two Trp residues: Trp-134 and Trp-213. The first one is located in the subdomain IA, on the surface. The second is located in subdomain IIA, within a hydrophobic pocket of BSA. In the structure of α-1-acid glycoprotein, there are three Trp residues: Trp-25, deep inside the β-barrel; Trp-122, near the entrance to the drug-binding pocket; and Trp-166, located on the surface of the protein [18,19]. In the structure of human gamma globulin, there are 20 Trp residues [20]. The intensity of BSA, AAG, and GG fluorescence was reducing with the increase in the phthalimide compound concentration in the sample, the results of which confirm the interaction between mixed components. This observation in quenching spectra of proteins BSA, AAG, and GG after the addition of compound A is presented in Figure 9 (quenching spectra of proteins: BSA, AAG, and GG after the addition of compound B-C are presented in Figure S1 in Supplementary Files). For all proteins, fluorescence intensity decreases regularly. Additionally, a shift in maximum emission is detected. Quenching in the fluorescence intensity of BSA is red-shifted. For interactions with AAG and GG, a blue shift occurred. This observation implies that the microenvironment around the chromophore of proteins is changed. The bathochromic shift (red) may indicate that the conformation of BSA was changed, and the amino acid residues are in a more polar environment. The hypsochromic shift (blue) for interactions with AAG and GG may mean that the amino acid residues are located in a more hydrophobic environment and are less exposed to the solvent [21]. Similar results were obtained for compounds B, C, and D. 

#### 3.2.3. Quenching Mechanism Analysis

The quenching mechanisms of BSA, AAG, or GG fluorescence in the presence of studied compounds could be either static or dynamic. To determine the quenching mechanism, the studies were conducted at three temperatures: 297, 303, and 308 K. The obtained data were processed according to the Stern–Volmer Equation (1). The inner filter effect was corrected using Equation (2):

(1) $$\frac{F_{0}}{F}=1+k_{q}\tau \left[Q\right]=1+K_{SV},\;$$

where *F*_0_ represents protein fluorescence intensity, *F* represents protein fluorescence intensities in with quencher, *k_q_* is the quenching rate constant, *τ*_0_ the average fluorescence lifetime of the biomolecule, [*Q*] is the quencher concentration, and *K_sv_* is the Stern–Volmer constant;

(2) $$F=F_{obs}10^{\frac{\left(A_{ex}+A_{em}\right)}{2}},$$

where *F* and *F_obs_* are the corrected and observed fluorescence intensities, respectively, *A_ex_* and *A_em_* are the absorbance values at excitation and emission wavelengths. 

**Figure 10:**
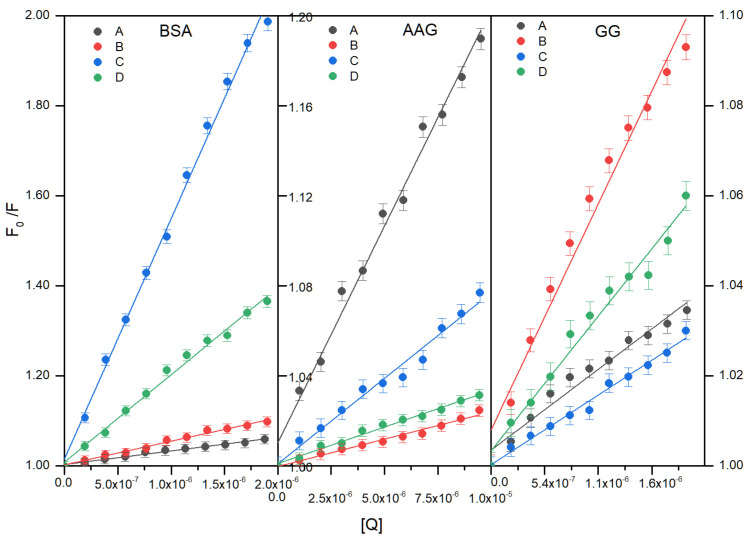

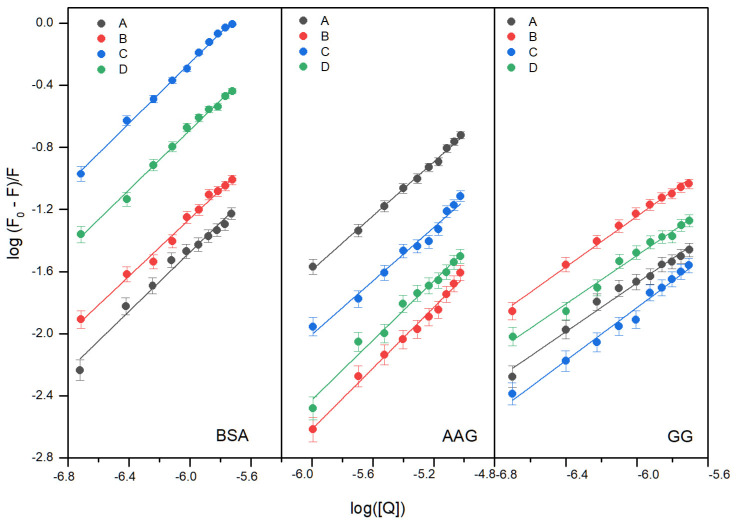
The Stern–Volmer regression plots (top) double logarithm plots (bottom) for quenching of BSA, AAG, and GG caused by the additive A–D compounds.

The experimental data were linear fitting and the Stern–Volmer (*K_SV_*) constant was determined (Figure 10). The quenching rate constant was also calculated with the use of the average lifetime (*τ*_0_) equal to 6 ns for all proteins [22,23]. For this purpose, the value of the Stern-–Volmer constant obtained was divided by the average lifetime. The results are given in Table 2, Table 3 and Table 4.

For dynamic quenching, the maximum value of the quenching rate constant in an aqueous solution is 2 × 10^10^ L·mol^−1^·s^−1^ [16,24]. The obtained *k_q_* values are much higher than 2 × 10^10^ L·mol^−1^·s^−1^ for all interactions with BSA, AAG, and GG. It suggests that a stable complex is forming. The calculated *k_q_* and *K_SV_* (Table 2) are the highest for complexes with BSA, indicating that studied compounds have a stronger affinity towards the excited fluorophores of BSA than those of other proteins. To verify the static quenching mechanism, fluorescence quenching experiments were performed at three different temperatures: 297, 303, and 308 K. The obtained results are presented in Table 2, Table 3 and Table 4. The *K_SV_* and *k_q_* values decrease with increasing temperature. This result confirms that interaction for analyzed compounds with BSA, AAG, and GG has a static mechanism.

#### 3.2.4. Binding Constant and Thermodynamic Parameters

To define the binding constants (*K_b_*) and the number of binding sites (*n*), double log regression curve (3) was used:

(3) $$log\frac{F_{0}-F}{F}=logK_{b}+nlog\left[Q\right].$$

As it is shown in Figure 10, a good linear relationship between log [(*F*_0_ − *F*)/*F*] and *log* [*Q*] is observed. The calculated results are collected in Table 2, Table 3 and Table 4. For the interactions with BSA, *K_b_* values were determined as 1.89 × 10^4^ (for A) to 4.36 × 10^5^ L·mol^−1^ (for C). The addition of the -OCH_3_ group into the ortho position in the phenyl does not affect the value of the *K_b_*. Structural modification of compounds C and D makes the complex with BSA more stable. The number of the binding site is close to 1, which shows one-to-one interaction. The double logarithm plot (Figure 10) is a linearization of the Hill equation. The coefficients n (Table 2, Table 3 and Table 4) for all studied interactions are lower than 1, suggesting a negative cooperative binding process [25,26,27]. The binding of one molecule to a protein reduces the affinity for binding to another site in the protein. As previous studies have shown, the four phthalimide derivatives studied in this work show good anti-inflammatory properties [3]. The interaction with albumin of drugs with such an effect was studied by Mohammadnia [28]. The determined binding constants are within a wide range: from 10^2^ L·mol^−1^ for acetaminophen to 1.88 × 10^7^ L·mol^−1^ for meloxicam. Therefore, the *K_b_* values of studied compounds show that the interactions with BSA are moderate. Similar values were obtained for many compounds with biological activity [29,30,31,32,33,34,35]. 

The obtained *K_b_* values for interactions with AAG and GG are lower than for the interactions with BSA (Table 2, Table 3 and Table 4). All structural modifications reduce the stability. For the complexes with GG, the *K_b_* values are similar and differ only slightly from the values for the interactions with AAG. For complexes with AAG, the highest value was determined for compound A—4.27 × 10^3^ L·mol^−1^. The constant obtained for the interaction with GG is smaller. On the other hand, the value for *K_b_* for compound B with GG is equal to 4.47 × 10^3^ L·mol^−1^ and is higher than in the case of AAG. For ligands C and D, the interaction with both proteins is similar, and the observed *K_b_* values differ slightly. The results indicate that formed complexes with AAG and GG are weaker and both proteins could be entangled in the delivery of A–D in the blood to a smaller degree. It should also be noted that lower values of the *K_b_* mean a less stable complex, i.e., easier release of the drug. Ultimately, the formation of a complex with all selected plasma proteins promotes the pharmacological efficacy of the drug.

The non-covalent forces involved in the interactions with proteins can be identified by the thermodynamics parameters such as enthalpy change (Δ*H*°), entropic change (Δ*S*°), and free energy change (Δ*G*°). Thermodynamic parameters were computed using Equations (4) and (5):

(4) $$logK_{b}=-\frac{\Delta H^{{}^{\circ}}}{RT}+\frac{\Delta S^{{}^{\circ}}}{R},$$

(5) $$\Delta G^{{}^{\circ}}=\Delta H^{{}^{\circ}}-T\Delta S^{{}^{\circ}}=-RTlnK_{b},$$

where *K_b_* represents the binding constant, and *R* represents the gas constant. The results are given in Table 2, Table 3 and Table 4. For all interactions, the Δ*G*° values are negative. It indicates that the binding process is spontaneous. The calculated values for Δ*H*° and Δ*S*° are also negative, indicating that distance-dependent contacts and hydrogen bonds are the main interaction types.

In the BSA molecule, there are two binding sites with a high affinity for drug binding. Site I is situated in subdomains IIA and site II is situated in subdomains IIIA [15]. To determine the binding sites where tested compounds can be bound, Phenylbutazone (PHB) and Ibuprofen (IBP) were used to identify binding sites. Site I shows the binding affinity towards PHB, site II is known to bind IBP [36]. Results indicate that the binding constant for interactions with all tested compounds decreased in the presence of both PHB and IBP markers (Table 5). However, for PHB, the differences are smaller than in the presence of IBP. It shows that the tested compounds can anchor in both subdomains. However, it seems that drug site II is more favored, which was also confirmed by a molecular modeling study.

### 3.3. Circular Dichroism Spectroscopy

**Figure 11:**
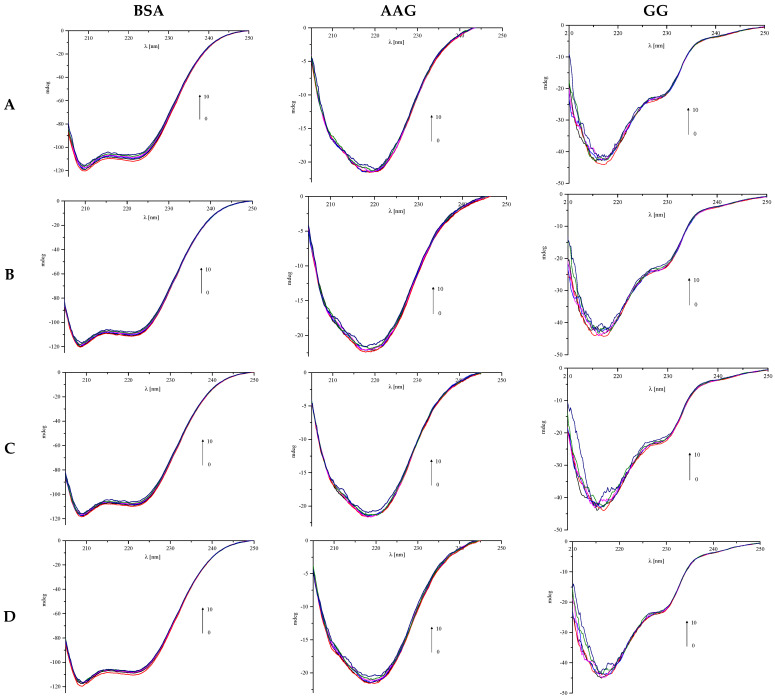
CD spectra of proteins: BSA, AAG, and GG after adding the appropriate portions of the tested ligands A, B, C, and D.

CD spectroscopy is a very useful method to study the secondary and tertiary structure of proteins [37]. Protein structures such as α-helix or β-sheet have characteristic bands on the CD spectrum. The first structure manifested by the presence of two negative bands near 209 and 220 nm. Meanwhile, the latter form is characterized by a band around 215 nm [38]. The formation of a complex between the protein and the tested compound can cause changes in the secondary structure, which are observed as changes in the recorded CD spectra. Therefore, we observed changes in the CD spectrum of the protein after adding the appropriate portions of the tested ligands to the solution to obtain the desired molar ratios, from 1:0 to 1:10. (Figure 11). Obtained results were analyzed by the CD Multivariate SSE program to determine the contents of the secondary structure components of proteins, and they are summarized in Table 6 and Table 7.

The measured spectra of circular dichroism are characteristic of the analyzed proteins. For serum albumin, two negative bands characteristic of the α-helix structure are observed (Figure 11) near 209 and 220 nm. An increase in the concentration of the analyzed phthalimide derivatives in the solution causes the reduction in the intensity of peaks. Thus, the tested compounds interact with the albumin molecule, which confirms the results obtained from fluorescence spectroscopy and theoretical calculations. However, their presence does not destabilize the BSA structure. In the last recorded spectrum, the alpha-helix is still the dominant form of the protein (Table 6). The changes in the α-helix content are the greatest for compound A (2.6%), and the least significant for D (1.4%).

AAG spectra have one negative band near 220 nm (Figure 11). The addition of each successive portion of the analyzed phthalimide derivatives slightly affects the course of the spectrum. It is also confirmed by the analysis of CD results by the CD Multivariate SSE program. As it has been shown in Table 7, the protein molecule consists mainly of α-helix and β-sheet, whose content is at the level of 30%. The number of particular forms does not change much with the increasing concentration of phthalimide analogs. Changes are a maximum of 2% (for α-helix of compound D) or less. Summarizing, the above results indicate that the studied ligands do not destabilize the AAG secondary structure.

The most significant changes in the spectra appearing with increasing concentrations of phthalimide derivatives are observed in the case of GG (Figure 11). Increasing noise is observed in each subsequent spectrum. Therefore, the percentage analysis of the individual secondary forms was omitted in this case. However, it is visible that the tested derivatives have the greatest effect on the secondary structure of the gamma globulin among all the studied proteins.

### 3.4. FT-IR Spectroscopy

**Figure 12:**
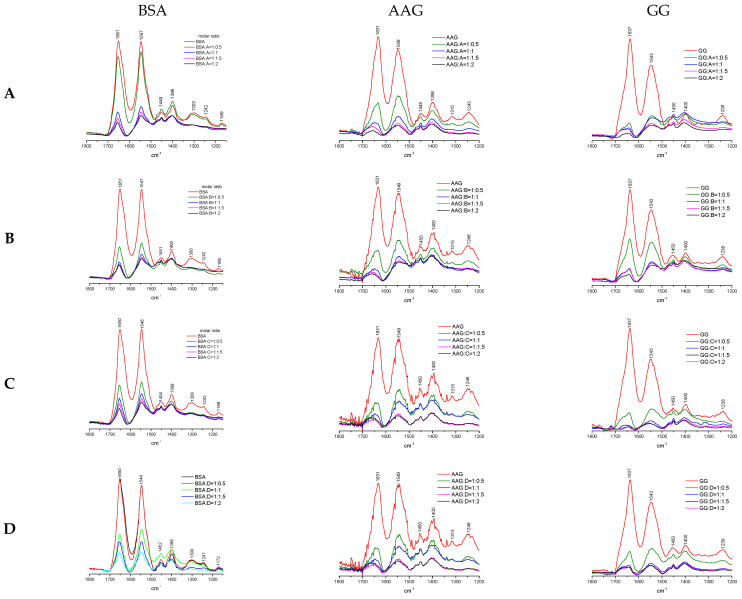
The changes in crucial spectral range contribution by interactions between proteins BSA, GG, AAG, and compounds A, B, C, and D with different molar ratios.

The infrared spectroscopy (IR) method combined with the mathematical algorithm of Fourier self-deconvolution (FSD) is wildly used to study the conformational changes in secondary structures of proteins. This approach was extensively utilized to analyze many biological fluids. The characteristic vibration of chemical groups in peptides and proteins reveal particular transmittance/absorption bands in the spectrum, namely Amide A (~3300 cm^−1^), Amide B (~3100 cm^−1^), and Amide I–VII (with respective positions ~1650 cm^−1^, ~1550 cm^−1^, ~1300 cm^−1^, ~735 cm^−1^, ~635 cm^−1^, ~600 cm^−1^, ~200 cm^−1^) [39,40,41]. Amide A and B signals originate from NH stretching vibration. The Amide I peak appears, first of all, as a result of stretching the C=O group, but also with contribution C–N stretching, C–C–N deformation, and N–H bending in-plane, whereas Amide II is caused mainly by N–H bending in-plane, and additionally by C–N stretching with contribution C–O bending in-plane and C–C and N–C stretching [39,41,42]. The secondary structure of a protein is related to the spatial arrangement of the peptide bonds chain and also interaction with adjacent amino acid residues in the sequence. However, Amide I is the most sensitive and the most conventional band to verify the conformation changes in protein [43,44]. It chiefly depends on peptide bond vibrations. The remaining Amide bands (III-VII) are not so important and useful in the analysis of the conformational structure of proteins because they mainly result from side-chain interactions of individual amino acids and other weak interactions such as hydrogen bonds. The free serum blood proteins were characterized by a unique IR spectrum, in which most variable regions 1800–1200 cm^−1^ are presented in Figure 12. The Amide I band for examined BSA, AAG, and GG was observed at 1651 cm^−1^, 1631 cm^−1^, and 1637 cm^−1^, respectively. In addition, the Amide II signal exhibits high intensity peak at position 1547 cm^−1^ for BSA, 1549 cm^−1^ for AAG, and 1543 cm^−1^ for GG. In contrast, the Amide III band is only observed for BSA at 1300 cm^−1^ is very weak for AAG at 1315 cm^−1^, and absent in the GG spectrum. The study of the interaction of phthalimide analogs with individual proteins was carried out by monitoring the changes in the peak intensity of Amide I and Amide II in various molar ratios. The general conclusion is the increase in the concentration of the compounds caused a significant decrease in the intensity and shape of the Amide I and II bands. A slight shift in the peaks was also observed as shown in Figure 12. The most spectacular changes occurred until an equimolar amount of protein and ligand was achieved. The compounds A and D did not exert any significant effect on the Amide bands of BSA after the addition of the first portion, unlike compounds B and C. On the other hand, in the interaction with GG, a drastic reduction in the absorption of these bands was observed. The result of a decreased intensity of peaks assumes the variation in the protein secondary structure. The next step was to bring under quantitative analysis Amide I peak for spectra with equimolar amount of compounds and proteins.

**Figure 13:**
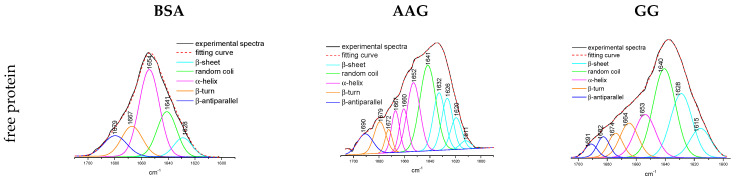

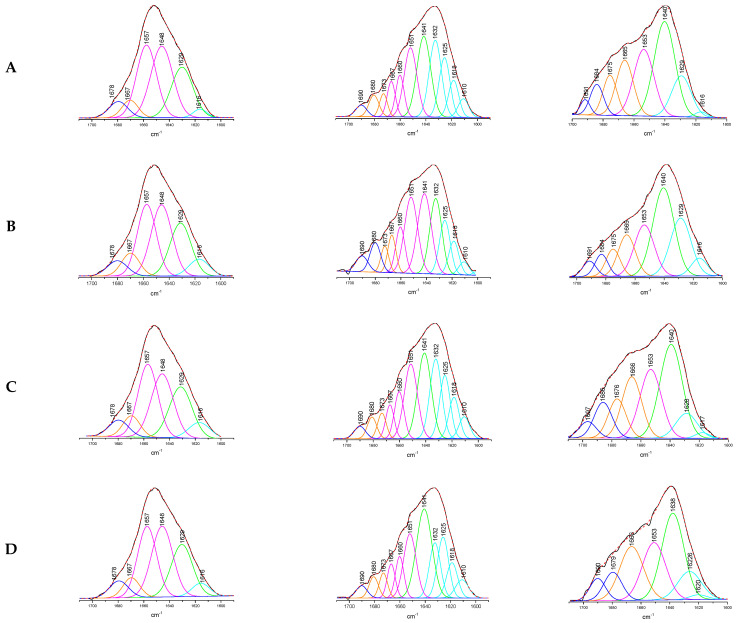
Distribution fitting curves of Amide I peak of the unbound proteins BSA, AAG, and GG and after interaction with phthalimide A, B, C, and D with equimolar ratio.

Moreover, the FT-IR spectra were brought under analysis according to the procedure by Byler and Susi [43]. The fragment with the Amide I peak at 1650 cm^−1^ was extracted after the normalization of each spectrum. The major peaks were selected from the second derivate. The signal position is correlated with the following structures: α-helix (1660–1650 cm^−1^), β-sheet (1640–1610 cm^−1^), β-turn (1691–1680 cm^−1^), β-antiparallel (1660–1650 cm^−1^) and random coil (1650–1640 cm^−1^) structures [39,40,41]. The self-deconvolution was conducted on the Amide I bands. The Gaussian function curve-fitting was performed, and the total area under the peak was determined. The percentage of area under peaks corresponds with the contribution of the secondary structure of the protein. In this work, the monitoring of detailed changes in the Amide I band was presented in Table 8 and Figure 13. 

The binding of phthalimides to BSA caused few-percent reductions in α-helix share in the protein structure for all studied derivatives. The highest influence was noticed for compounds B with the -OCH_3_ group and C with a substituent -CF_3_. Moreover, a slight decrease in β-antiparallel structure was detected. As a result, the contribution mostly to β-sheet, but also to β-turn and unstructured random conformation has increased. In the case of interaction with AAG, compounds B and C also showed the greatest destabilization of the α-helix. At the same time, the share of the antiparallel β-sheet decreased, which may be related to the breach with the hydrogen bonding network in the protein structure. The increase in percentage contribution in β-sheet and β-turn was evaluated for all derivatives, but the most was observed for A. The greatest variation in secondary structure was detected for complexes with the GG. The interaction between protein and phthalimide compounds caused a decrease in the percentage of the β-turn structure in favor of the α-helix, β-turn, and β-antiparallel share. The random coil remained at a similar level without much influence. 

The study of the secondary structure of serum blood proteins was analyzed by two instrumental techniques: circular dichroism spectroscopy (CD) and infrared spectroscopy (FT-IR). The results of both methods have shown a similar tendency in changes in the secondary structure after bounding investigated phthalimide derivatives, but the data acquired are slightly different. Those disagreements may result from no identical conditions, such as not having the same concentrations. Another reason is the different mathematical algorithms that were used to analyze data. However, the results obtained from both experiment methods are consistent and lead to the same overall conclusions.

## 4. Conclusions

In this work, we showed the analysis of the interaction between four non-toxic phthalimide derivatives and three plasma proteins: serum albumin, gamma globulin, and α1-acid glycoprotein. We used several spectroscopic methods: fluorescence, circular dichroism, and FT-IR. Additionally, the results obtained with the use of experimental methods were confirmed and visualized using molecular docking. 

Analyzed compounds A, B, and C are characterized by their own fluorescence. Therefore, a 300 nm excitation wavelength was used. This is rather unusual for protein excitation wavelength allowed to analyze the changes occurring in the fluorescence phenomenon and at the same time avoid overlapping of the effect originating from the protein molecule and the tested compounds. The emission band in fluorescence spectroscopy decreases with increasing concentration of phthalimide derivatives. The mechanism of quenching is static for all analyzed systems and ground-state complexes are formed between protein and analyzed compound molecules. All analyzed proteins bind one molecule of each of the analyzed compounds, which is confirmed by a molecular docking study. Compounds can be bound by BSA through two possible sites, I or II, depending on availability. However, the molecular docking along with fluorescence competitive binding PHE and IBP measurements suggested that site II is preferable. Compounds C and D formed more stable complexes with BSA than A and B. The addition of the -OCH_3_ group into the ortho position in the phenyl in the B molecule does not affect the value of the *K_b_* compared to compound A. The observed interactions between studied ligands and AAG and GG are weaker than those for albumin. However, for complexes with AAG, the highest value of *K_b_* was detected for compound A. All structural modifications reduce stability. For the complexes with GG, the *K_b_* values are similar to those with AAG. The complex formed with ligand B shows the greatest stability here. For the tested compounds C and D, the binding constants with AAG and GG proteins are of similar value. The obtained results show that the AAG and GG proteins could be involved in the transport of the tested compounds A–D to a much lesser extent than albumin. It is worth noting, however, that a lower *K_b_* value may also mean easier displacement of compounds from the complex with the protein, thus increasing the concentration of the active form of potential pharmaceuticals. In summary, the binding of the test compounds to plasma components promotes the pharmacological efficacy of the drug. Furthermore, results from CD and FT-IR spectroscopies showed that the interaction between analyzed molecules and plasma proteins did not affect the structures of BSA and AAG. However, in the case of GG, a large influence of the presence of phthalimide derivatives on the secondary structure of the protein molecule was observed. The presented results confirm the pharmaceutical potential of phthalimide derivatives.

## Figures and Tables

**Table 1:** The values of Δ*G*°, Binding Free Energy (AutoDock scoring function) for interaction phthalimides A–D with Bovine Serum Albumin (BSA), Human Serum Albumin (HSA), α1-acid glycoprotein (AAG), and gamma globulin (GG).

	Binding Free Energy Δ*G*° [kJ·mol^−1^]					
	BSA		HSA		AAG	GG
	site I	site II	site I	site II		
A	−32.22	−34.39	−35.27	−35.32	−31.64	−33.83
B	−31.89	−32.69	−34.56	−37.45	−33.23	−31.90
C	−27.42	−30.85	−34.98	−38.71	−33.18	−35.09
D	−34.19	−37.91	−41.30	−43.18	−37.62	−35.12

**Table 2:** The fluorescence parameters: *K_sv_* (the Stern–Volmer constant), *k_q_* (the quenching rate constant), n (a number of binding sites) at temperatures 297, 303, and 308 K and thermodynamic quantities: Δ*G*°, Δ*H*°, Δ*S*° for interaction between BSA and studied compounds.

		Quenching Constants		Binding Parameters			Thermodynamic Parameters		
	T [K]	*K_sv_* × 10^4^ [L·mol^−1^]	*k_q_* × 10^12^ [L·mol^−1^·s^−1^]	*logK_b_*	*K_b_* × 10^4^ [L·mol^−1^]	n	Δ*G*° [kJ·mol^−1^]	Δ*H*° [kJ·mol^−1^]	Δ*S*° [J·mol^−1^K^−1^]
A	297 303 308	3.00 ± 0.12 1.68 ± 0.07 0.83 ± 0.04	5.00 2.80 1.38	4.27 ± 0.22 3.87 ± 0.23 3.22 ± 0.20	1.89 0.74 0.16	0.96 ± 0.04 0.94 ± 0.04 0.88 ± 0.03	−24.66	−166.58	−477.89
B	297 303 308	5.24 ± 0.17 4.68 ± 0.16 2.87 ± 0.13	8.73 7.80 4.78	4.30 ± 0.20 4.06 ± 0.13 3.57 ± 0.21	2.01 1.14 0.37	0.93 ± 0.03 0.89 ± 0.02 0.84 ± 0.04	−24.73	−114.32	−301.63
C	297 303 308	53.42 ± 1.12 38.09 ± 0.86 24.64 ± 0.46	89.00 63.50 41.10	5.64 ± 0.12 5.19 ± 0.18 4.84 ± 0.16	43.61 15.65 6.95	0.98 ± 0.02 0.93 ± 0.03 0.90 ± 0.03	−32.06	−127.43	−321.09
D	297 303 308	19.40 ± 0.57 15.62 ± 0.28 9.63 ± 0.15	32.30 26.00 16.10	5.07 ± 0.16 4.89 ± 0.09 4.67 ± 0.06	11.72 7.78 4.67	0.96 ± 0.03 0.95 ± 0.01 0.94 ± 0.01	−28.90	−63.19	−115.48

**Table 3:** The fluorescence parameters: *K_sv_* (the Stern–Volmer constant), *k_q_* (the quenching rate constant), n (a number of binding sites) at temperatures 297, 303, and 308 K and thermodynamic quantities: Δ*G*°, Δ*H*°, Δ*S*° for interaction between AAG and studied compounds.

		Quenching Constants		Binding Parameters			Thermodynamic Parameters		
	T [K]	*K_sv_* × 10^4^ [L·mol^−1^]	*k_q_* × 10^12^ [L·mol^−1^·s^−1^]	*logK_b_*	*K_b_* × 10^3^ [L·mol^−1^]	n	Δ*G*° [kJ·mol^−1^]	Δ*H*° [kJ·mol^−1^]	Δ*S*° [J·mol^−1^K^−1^]
A	297 303 308	1.93 ± 0.10 1.90 ± 0.12 1.87 ± 0.18	3.22 3.17 3.12	3.63 ± 0.09 3.06 ± 0.20 2.89 ± 0.20	4.27 1.15 0.77	0.87 ± 0.02 0.76 ± 0.04 0.72 ± 0.04	−20.37	−119.64	−334.22
B	297 303 308	0.24 ± 0.02 0.23 ± 0.03 0.23 ± 0.03	0.40 0.38 0.38	3.27 ± 0.19 2.71 ± 0.12 2.16 ± 0.15	1.86 0.51 0.15	0.98 ± 0.04 0.87 ± 0.02 0.75 ± 0.03	−18.68	−176.19	−530.35
C	297 303 308	0.76 ± 0.04 0.73 ± 0.04 0.72 ± 0.05	1.27 1.22 1.20	3.17 ± 0.24 2.55 ± 0.25 2.04 ± 0.18	1.48 0.32 0.11	0.86 ± 0.05 0.74 ± 0.05 0.64 ± 0.03	−18.05	−179.82	−544.65
D	297 303 308	0.33 ± 0.02 0.32 ± 0.03 0.31 ± 0.04	0.55 0.53 0.52	3.32 ± 0.25 2.63 ± 0.29 1.93 ± 0.30	2.09 0.43 0.08	0.96 ± 0.05 0.82 ± 0.06 0.68 ± 0.06	−19.01	−221.88	−683.07

**Table 4:** The fluorescence parameters: *K_sv_* (the Stern–Volmer constant), *k_q_* (the quenching rate constant), n (a number of binding sites) at temperatures 297, 303, and 308 K and thermodynamic quantities: Δ*G*°, Δ*H*°, Δ*S*° for interaction between GG and studied compounds.

		Quenching Constants		Binding Parameters			Thermodynamic Parameters		
	T [K]	*K_sv_* × 10^4^ [L·mol^−1^]	*k_q_* × 10^12^ [L·mol^−1^·s^−1^]	*logK_b_*	*K_b_* × 10^3^ [L·mol^−1^]	n	Δ*G*° [kJ·mol^−1^]	Δ*H*° [kJ·mol^−1^]	Δ*S*° [J·mol^−1^K^−1^]
A	297 303 308	1.66 ± 0.09 1.22 ± 0.10 1.20 ± 0.14	2.77 2.03 2.00	3.04 ± 0.22 2.41 ± 0.18 1.68 ± 0.40	1.10 0.26 0.05	0.79 ± 0.04 0.71 ± 0.03 0.58 ± 0.07	−17.51	−215.08	−665.20
B	297 303 308	4.63 ± 0.24 4.56 ± 0.34 4.47 ± 0.43	7.72 7.60 7.45	3.65 ± 0.15 3.01 ± 0.36 2.60 ± 0.27	4.47 1.02 0.40	0.81 ± 0.03 0.72 ± 0.06 0.64 ± 0.05	−20.67	−169.35	−500.59
C	297 303 308	1.38 ± 0.05 1.34 ± 0.07 1.31 ± 0.10	2.30 2.23 2.18	3.22 ± 0.27 2.77 ± 0.18 2.39 ± 0.17	1.58 0.59 0.25	0.84 ± 0.05 0.76 ± 0.03 0.70 ± 0.03	−18.34	−131.90	−382.36
D	297 303 308	2.61 ± 0.14 1.43 ± 0.07 1.29 ± 0.08	4.35 2.38 2.15	3.19 ± 0.20 2.80 ± 0.31 2.37 ± 0.33	1.55 0.63 0.23	0.78 ± 0.03 0.76 ± 0.06 0.70 ± 0.06	−18.24	−129.80	−375.63

**Table 5:** The binding constant of the phthalimide compounds A–D with BSA/PHB and BSA/IBP complexes at 297 K.

Complex and Binding Site	*logK_b_*			
	A	B	C	D
- BSA + PHB (site I) BSA + IBP (site II)	4.27 ± 0.22 2.98 ± 0.18 2.52 ± 0.19	4.30 ± 0.20 3.04 ± 0.15 2.62 ± 0.13	5.64 ± 0.12 3.94 ± 0.18 3.27 ± 0.09	5.07 ± 0.16 3.42 ± 0.10 3.07 ± 0.11

**Table 6:** The percentage of each secondary structure form for bovine serum albumin after adding the appropriate portions of the tested ligands A, B, C, and D.

BSA: Analyzed Compound Molar Ratio	% α-Helix	% β-Sheet	% Turn	% Other
Compound A				
1:0	59.7%	5.3%	10.0%	25.0%
1:0.5	58.9%	5.8%	10.0%	25.3%
1:1	58.4%	6.2%	10.0%	25.3%
1:2	58.0%	6.4%	10.1%	25.5%
1:5	57.5%	6.7%	10.1%	25.6%
1:10	57.1%	7.7%	10.1%	25.2%
Compound B				
1:0	59.5%	5.5%	10.0%	25.0%
1:0.5	59.3%	5.6%	10.0%	25.2%
1:1	59.0%	5.9%	10.0%	25.1%
1:2	58.6%	6.2%	10.0%	25.2%
1:5	58.3%	6.3%	10.1%	25.4%
1:10	57.8%	6.6%	10.1%	25.5%
Compound C				
1:0	58.7%	5.9%	10.1%	25.4%
1:0.5	58.2%	6.3%	10.1%	25.4%
1:1	57.8%	6.3%	10.1%	25.7%
1:2	57.7%	6.7%	10.1%	25.5%
1:5	57.4%	6.8%	10.1%	25.7%
1:10	57.0%	7.5%	10.1%	25.4%
Compound D				
1:0	58.9%	5.9%	10.0%	25.2%
1:0.5	58.1%	6.4%	10.1%	25.5%
1:1	57.9%	7.0%	10.0%	25.1%
1:2	57.7%	6.7%	10.1%	25.5%
1:5	57.6%	7.3%	10.1%	25.1%
1:10	57.5%	7.3%	10.1%	25.2%

**Table 7:** The percentage of each secondary structure form for α1-acid glycoprotein after adding the appropriate portions of the tested ligands A, B, C, and D.

AAG: Analyzed Compound Molar Ratio	% α-Helix	% β-Sheet	% Turn	% Other
Compound A				
1:0	32.8%	29.6%	9.6%	27.9%
1:0.5	32.8%	29.9%	9.6%	27.7%
1:1	32.6%	29.9%	9.6%	27.9%
1:2	32.5%	29.9%	9.6%	28.0%
1:5	32.1%	30.0%	9.7%	28.3%
1:10	31.8%	30.3%	9.7%	28.2%
Compound B				
1:0	34.6%	29.3%	9.5%	26.6%
1:0.5	34.4%	29.6%	9.5%	26.5%
1:1	34.0%	29.5%	9.6%	26.9%
1:2	33.8%	29.6%	9.6%	27.1%
1:5	33.6%	30.0%	9.5%	26.9%
1:10	33.1%	30.0%	9.6%	27.3%
Compound C				
1:0	33.3%	29.7%	9.7%	27.4%
1:0.5	33.1%	29.9%	9.6%	27.4%
1:1	32.6%	30.0%	9.6%	27.9%
1:2	32.9%	30.0%	9.6%	27.5%
1:5	32.7%	30.0%	9.7%	27.6%
1:10	32.0%	29.9%	9.8%	28.3%
Compound D				
1:0	33.1%	29.7%	9.7%	27.6%
1:0.5	32.8%	29.9%	9.7%	27.6%
1:1	32.4%	29.9%	9.7%	28.1%
1:2	32.1%	30.0%	9.7%	28.2%
1:5	32.0%	30.2%	9.7%	28.1%
1:10	31.1%	30.1%	9.8%	29.0%

**Table 8:** The percentage of the secondary structure of major blood serum components BSA, AAG, GG, and complexes with phthalimides A, B, C, and D at pH = 7.5 with equimolar ratio, calculated from deconvolution Amide I band.

Protein or Protein/Phthalimide Complexes	The Percentage of Secondary Structure Component [%]				
	α-Helix	β-Sheet	β-Turn	β-Anti	Random
BSA					
unbound protein	60.40%	6.97%	9.35%	7.55%	15.73%
A	58.07%	8.30%	10.20%	6.11%	17.32%
B	57.92%	8.20%	10.11%	7.30%	16.47%
C	57.63%	8.18%	10.41%	7.46%	16.32%
D	58.10%	8.43%	10.32%	6.21%	16.94%
AAG					
unbound protein	29.04%	30.69%	7.62%	8.11%	24.54%
A	27.05%	36.51%	9.17%	6.46%	20.81%
B	26.54%	35.81%	8.67%	7.54%	21.44%
C	26.53%	35.87%	8.12%	7.72%	21.76%
D	27.10%	35.43%	8.62%	6.73%	22.12%
GG					
unbound protein	14.37%	31.70%	15.57%	6.80%	31.56%
A	21.52%	13.75%	24.16%	8.91%	31.66%
B	16.97%	24.93%	18.41%	8.30%	31.39%
C	21.07%	8.74%	27.09%	13.15%	29.94%
D	21.82%	12.21%	20.82%	13.99%	31.16%

## Data Availability

Data sharing not applicable.

## Supplementary Materials

The following supporting information can be downloaded at: https://www.mdpi.com/article/10.3390/life13030760/s1, Figure S1: Fluorescence spectra BSA, AAG, and GG in the presence of different concentrations of compound B, C and D.; Figure S2: The plane diagram of a type of interaction between phthalimide derivatives and Human Serum Albumin. On the left in site I, and on the right in site II.
