# Supplementary material for: Spectroscopic and Theoretical Analysis of the Interaction between Plasma Proteins and Phthalimide Analogs with Potential Medical Application
Source: Life (Basel). 2023 Mar 10;13(3):760. doi: 10.3390/life13030760 (PMC10051393; doi:10.3390/life13030760)
Supplement: Supplementary file 1 [file life-13-00760-s001.zip › life-2253584-supplementary.pdf]

Supplementary File of Spectroscopic and theoretical analysis of the interaction between plasma proteins and phthalimide analogs with potential medical application

Edward Krzyżak [1,*], Aleksandra Kotynia [1], Dominika Szkatuła [2], Aleksandra Marciniak [1,*]

[1] Department of Basic Chemical Sciences, Wroclaw Medical University, Borowska 211a, 50-556 Wrocław, Poland

[2] Department of Medicinal Chemistry, Wroclaw Medical University, Borowska 211, 50-556 Wrocław, Poland; dominika.szkatula@umw.edu.pl

* Correspondence: edward.krzyzak@umw.edu.pl (E.K.), aleksandra.marciniak@umw.edu.pl (A.M.) ; Tel.: +48 71 784 03 32 (A.M.).

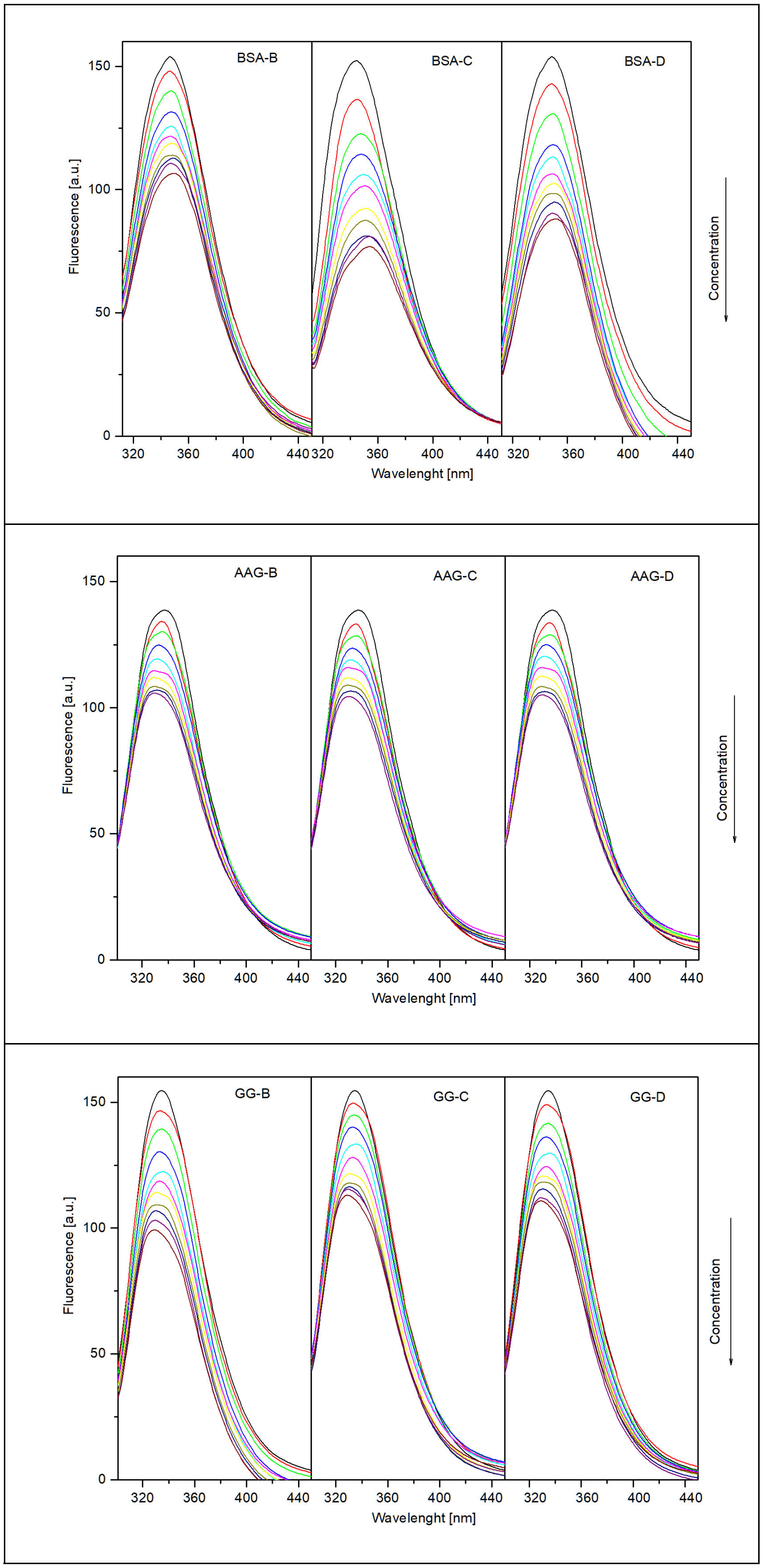

Figure S1. Fluorescence spectra BSA, AAG, and GG in the presence of different concentrations of compound B, C and D.

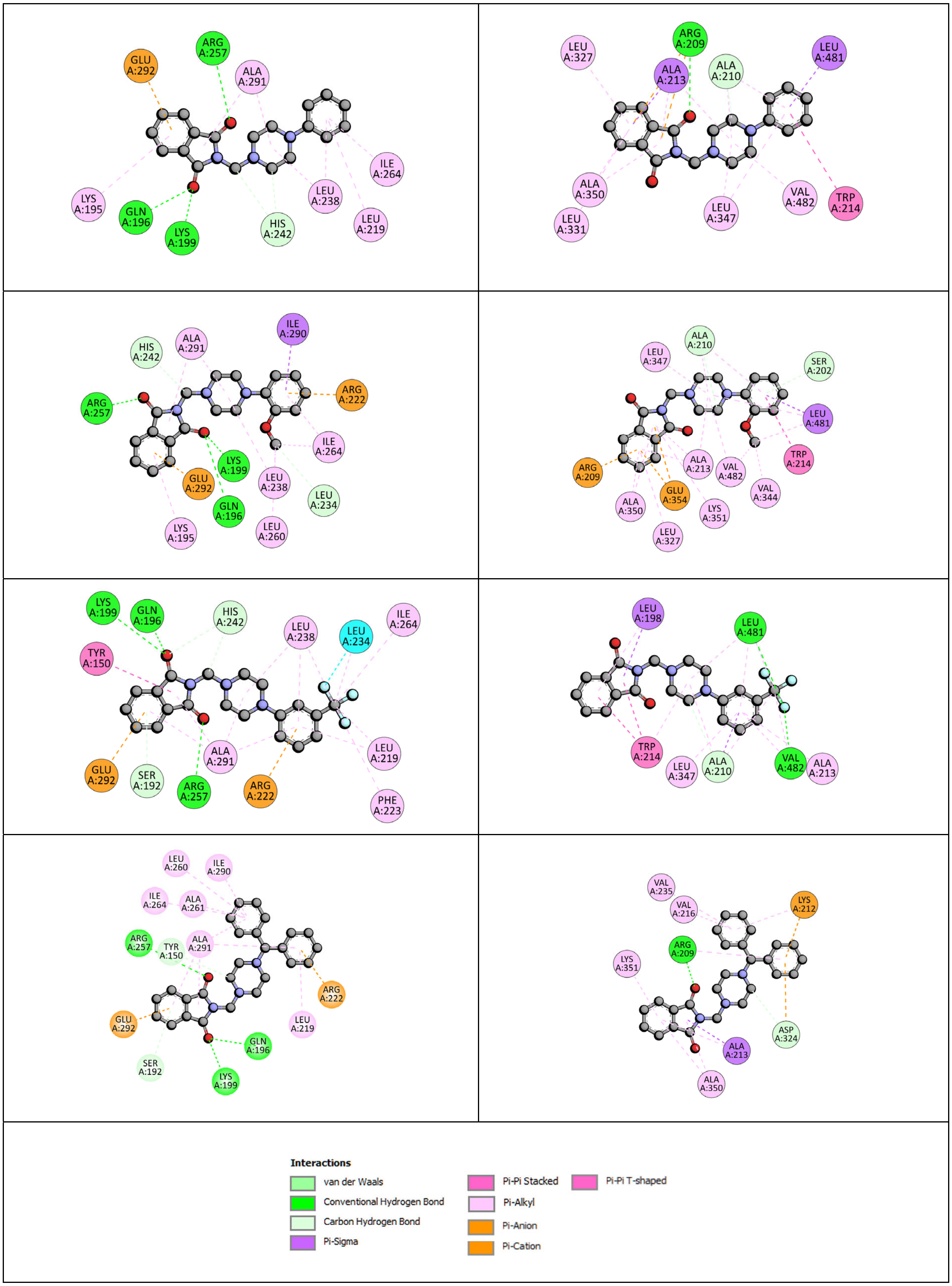

Figure S2. The plane diagram of a type of interaction between phthalimide derivatives and Human Serum Albumin. On the left in site I, and on the right in site II).
